# A Rare Case of Cavernous Haemangioma of the Adrenal Gland: A Case Report and Review of Literature

**DOI:** 10.7759/cureus.29917

**Published:** 2022-10-04

**Authors:** Yunli Chua, Sharmaine Quake, Kolanu Prasad, Wael Elsaify

**Affiliations:** 1 General Surgery, James Cook University Hospital, Middlesbrough, GBR; 2 Pathology, James Cook University Hospital, Middlesbrough, GBR

**Keywords:** adrenal cavernous haemangioma, rare adrenal tumour, adrenalectomy, incidentaloma, adrenal gland, cavernous haemangioma

## Abstract

Cavernous haemangiomas, also known as cavernoma or cavernous angiomas, are clusters of vasculature malformations arising from the endothelial layer of blood vessels. They are commonly found in the central nervous systems, skin, or liver. Rarely, they can also affect adrenal glands, a phenomenon with only 66 cases since the first case was reported in the literature in 1955 and 2018. Adrenal cavernous haemangiomas are typically non-functioning and found incidentally on radiological imaging.

Here, we present the case of a 79-year-old male who was referred by a district general hospital to our tertiary centre with an incidentaloma of the left adrenal gland which was first noted in 2014 measuring 6 cm. A repeat computed tomography in 2020 revealed the mass was 20.8 cm. In 2020, the growing mass was causing anaemia and abdominal discomfort due to displacement of the surrounding viscera.

The initial radiological impression performed in another hospital of the indeterminate mass was highly suspicious of primary adrenal malignancy. The decision was made to operate prior to biopsy as biopsy was deemed high risk for dissemination of primary malignancy of the adrenal.

The patient subsequently underwent a radical open left adrenalectomy in September 2020. The diagnosis of adrenal cavernous haemangioma was made on histopathological examination post-surgery. Published literature on this rare adrenal tumour between 2019 and 2021 is also reviewed in this paper.

## Introduction

Incidental adrenal masses, or incidentalomas, are asymptomatic adrenal tumours detected on radiological imaging as part of patients’ workup for other complaints unrelated to the described lesion [1]. The prevalence of adrenal incidentalomas is reported to be up to 10% on radiological studies in the elderly population [1].

Cavernous haemangiomas are clusters of vasculature malformations arising from the endothelial layer of blood vessels [2]. Very rarely, they can also affect adrenal glands [3]. Here, we present the case of an adrenal cavernous haemangioma diagnosed on histology after surgical management and review the literature published between 2019 and 2021 on this rare clinical entity.

## Case presentation

**Figure 1:**
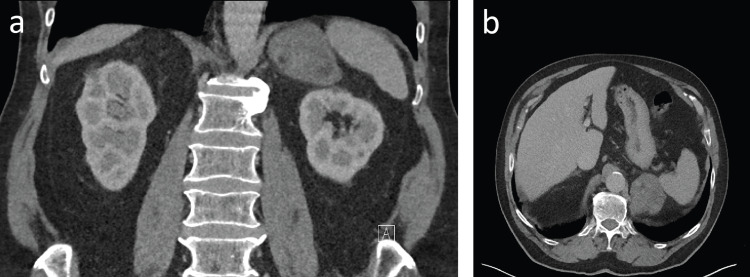
The index computed tomography scan in 2014. (a) Coronal view showing a left adrenal mass measuring 5.5 × 4.8 × 5.6 cm. (b) Axial view.

We report the case of a 79-year-old male with a left adrenal mass diagnosed incidentally in 2014 in the patient’s local hospital. The patient’s medical history included atrial fibrillation and longstanding multiple pulmonary nodules. He did not have any previous surgery. The index computed tomography (CT) scan in 2014 showed a left adrenal mass measuring 5.5 × 4.8 × 5.6 cm, containing foci of fat, necrosis, and calcification with a portal venous phase of 30 HU (Figures 1a, 1b).

The patient was counselled but declined further diagnostic and therapeutic intervention as he was asymptomatic. The patient continued to be under annual surveillance and interval imaging in his local hospital. In 2017, imaging revealed gradual progression in the size of the adrenal tumour up to 8 cm. The patient was aware that malignancy could not be excluded at least until a biopsy was done. However, he continued to decline investigation and intervention.

In March 2020, the patient again presented to his general practitioner with symptoms of intermittent pain in the left iliac fossa and anaemia, whereby his haemoglobin level was 70 g/L. There were no associated symptoms of bleeding, headaches, palpitations, and changes to his appetite or weight. On examination, there was a non-tender palpable mass in the left iliac fossa.

**Figure 2:**
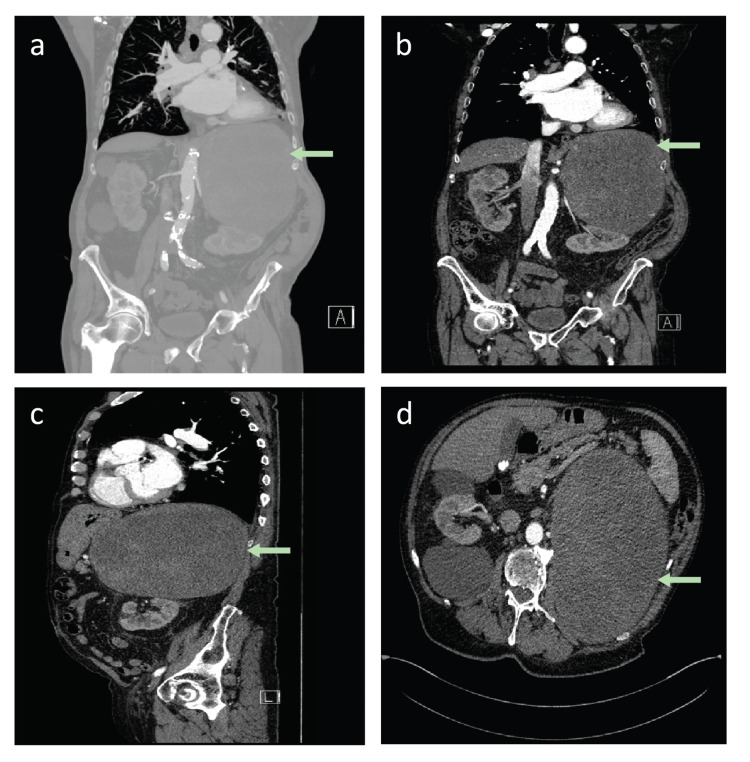
Computed tomography scan of the abdomen and pelvis in 2020. (a) Coronal view showing a large left suprarenal mass measuring 20.8 cm. (b) Coronal view showing the lesion was displacing the spleen, pancreas, left kidney, and in very close proximity to splenic renal vessels and the aorta. (c) Sagittal view. (d) Axial view.

A repeat CT scan in July 2020 (Figures 2a-2d) revealed a significant size increase of the left adrenal mass to 20.8 cm, causing displacement of the spleen, pancreas, left kidney, and in very close proximity to the splenorenal vessels and the aorta. There was no obvious invasion of adjacent structures (Figure 2b).

The preliminary biochemical tests prior to surgery suggested a non-functioning tumour. Taking into account the global clinical picture, the adrenal mass was more suspicious of a non-functional primary adrenal cancer and unlikely to represent metastases. The decision to operate prior to biopsy was due to the high risk of dissemination of malignancy in case it was a primary adrenal cancer.

Following the patient’s referral to our tertiary hospital, surgical management was recommended following multidisciplinary discussion with the aims of achieving a diagnosis, relieving the patient’s symptoms, and preventing complications such as spontaneous haemorrhage. This was also in line with the recommendation of the European Society of Endocrinology Clinical Practice Guideline on the management of adrenal incidentalomas [1].

**Figure 3:**
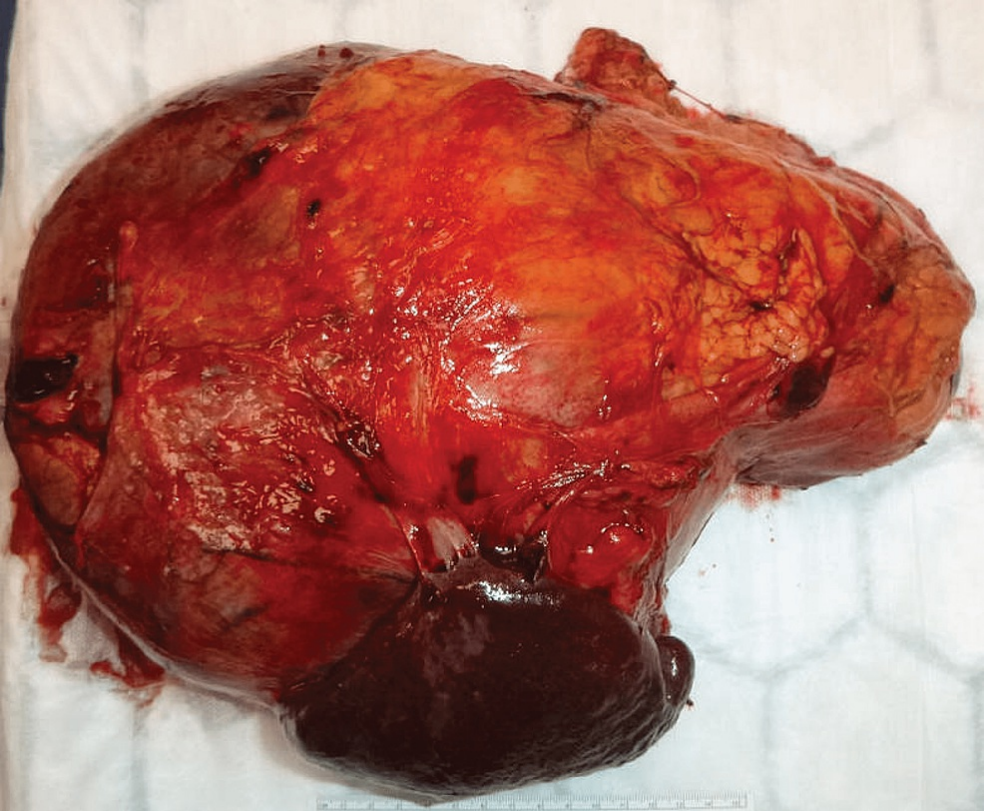
Resected en-bloc of the tumour.

The patient underwent an open radical left adrenalectomy where the en-bloc of the tumour was resected through a mid-line incision. The operative time was approximately six hours and 50 minutes, while intra-operative blood loss was approximately 400 mL. The patient had two units of packed red blood cells transfused prior to the procedure and was further transfused two units of packed red blood cells on day three post-operatively. Intra-operatively, the left adrenal tumour was found attached to the spleen, left kidney, and distal pancreas despite there being no invasion of those structures on the most recent radiological imaging. The specimen was subsequently removed en-bloc (Figure 3).

Chyle leak was diagnosed on day seven post-operatively from fluid in the patient’s left abdominal drain bag. The patient was managed conservatively with the use of somatostatin analogue (octreotide 100 µg three times a day) together with the dietitian’s modified low-fat diet. Apart from that, the patient’s recovery was uneventful and he was discharged home after 23 days of hospital admission. The chyle leak had resolved when the patient returned for his follow-up drain removal on day 27.

On gross pathological examination of the specimen, the encapsulated adrenal mass consisted of the adrenal with attached spleen, left kidney, and distal pancreas. The combined weight of the specimen was 4,060 g. The spleen and left kidney were both attached to the tumour. The encapsulated adrenal mass measured 18 × 13 × 7 cm.

**Figure 4:**
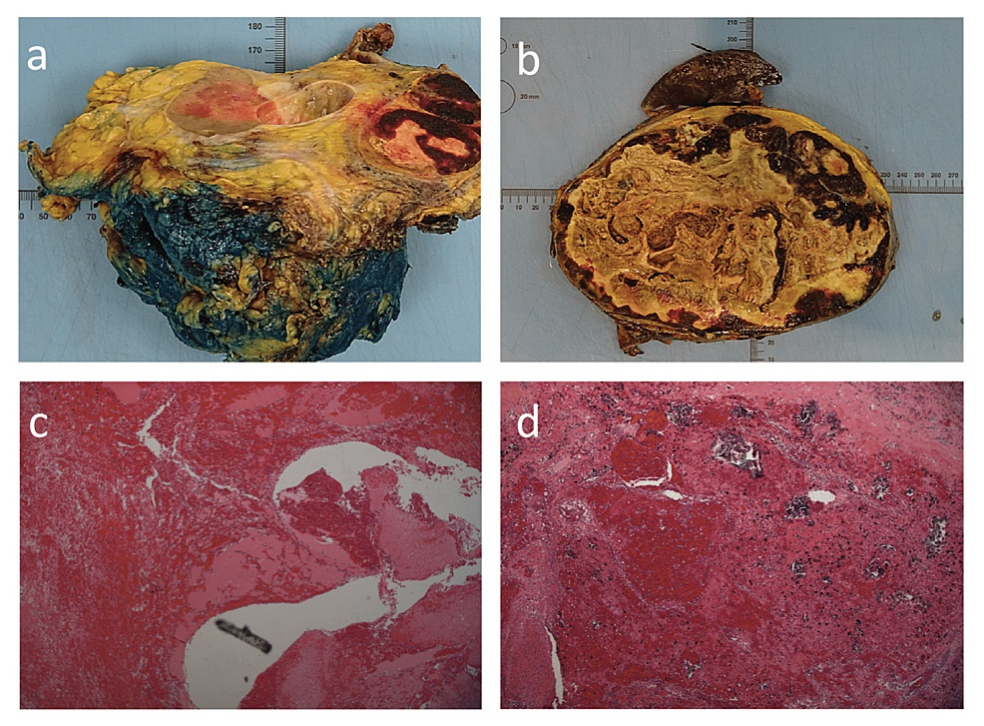
Histopathological images. (a) Resected mass dyed in ink. (b) Cross-section of the mass. (c) Microscopic image showing calcification. (d) Microscopic image showing haemangioma.

On slicing, most of the tumour appeared to consist of necrotic tissue around the edges, of which there was a dark haemorrhagic appearance (Figure 4, Panel b). There was some evidence of extension of the tumour beyond the capsule in the region of the upper pole of the left kidney where it was possibly infiltrating and/or arising from the adrenal gland.

The encapsulated tumour consisted largely of dilated blood vessels, many of which were filled with recent and organising thrombi. There were large areas of fibrosis and sclerosis with evidence of old haemorrhage (Figure 4, Panel d) in the form of collections of haemosiderophages as well as foci of dystrophic calcification.

In many areas, there was evidence of papillary endothelial hyperplasia of the cells lining many of the dilated blood vessels. The adrenal gland was identified outside the capsule of the lesion, and there was some evidence of atrophy as a result of compression by the tumour. The attached kidney, spleen, and distal tail of the pancreas showed no histological lesion. There was no evidence of malignancy.

## Discussion

Adrenal cavernous haemangiomas are rare, non-functioning, benign tumours which are commonly diagnosed with histological evidence following surgery, as seen in our patient. The first case of adrenal haemangioma in a live patient was reported by Johnson and Jeppesen [2] in 1955.

Between 1955 and 2018, only 66 cases have been reported in the literature, reflecting the rarity of this condition. In addition to the literature review by Degheili et al. [3], seven published cases between 2019 and 2021 were identified following a literature search using PubMed and Medline (Table 1). Together with our case, there were only 74 cases (Table 2) reported in the past 66 years.

**Table 1:** List of all published cases of adrenal cavernous haemangiomas between 2019 and 2021.

Case number	Authors (year of publication)	Age /gender	Laterality	Size (cm)	Presentation	Surgery approach	Speckled calcifications	Metabolic workup
1	Tica et al. (2019) [4]	68/F	Left	21 × 18.2 × 20 cm	Abdominal discomfort	Midline laparotomy	Infra-centrimetric calcifications	Not performed
2	Kansoun et al. (2020) [5]	70/F	Left	17 × 11 × 10 cm	Syncope and anaemia due to a bleeding adrenal haemangioma	Midline laparotomy	No data	Normal
3	Degheili et al. (2019) [3]	83/M	Right	8 × 7 × 3 cm	Vague abdominal bilateral flank pain	Right open radical adrenalectomy	No data	Normal
4	Jenkins et al. (2020) [6]	62/M	Right	9.5 cm	Right upper quadrant discomfort	Right open adrenalectomy and nephrectomy	No data	Normal
5	Gupta et al. (2020) [7]	79/M	Right	8 × 7 × 3.5 cm	Right flank pain, dull	Right open adrenalectomy	Punctate calcification	Normal
6	Al-Rawashdah et al. (2020) [8]	58/M	Left	7 × 6.7 × 6.1 cm	Left loin pain	Left laparoscopic adrenalectomy	Tiny calcification spots	Normal
7	Huang et al. (2021) [9]	67/M	Right	6.3 × 9.5 cm	Dull right back pain	Right laparoscopic adrenalectomy	No data	Normal

**Table 2:** Summary of characteristics of previously reported cases in the literature between 1955 and 2021. Data combined with Degheili et al. 2019 [3].

Characteristics	Data (N = 73)
Median age (year)	61.7
Sex	
Female	43
Male	30
Laterality	
Right	35 (48%)
Left	38 (52%)
Symptoms	
Asymptomatic	38 (52%)
Vague abdominal symptoms	12 (16.4%)
Flank pain	9 (12.3%)
Speckled calcifications	
Present	31 (47%)
Absent	32 (44%)
Metabolic workup	
Normal	51 (70%)
Abnormal	6 (8%)
Hyperaldosteronism	3 (4%)
Subclinical Cushing’s syndrome	3(4%)
Surgical approach	
Open	52 (71%)
Laparoscopic	18 (24.6%)

Over half of these tumours were initially detected incidentally on imaging as incidentalomas [10]. The majority of small adrenal lesions, in particular, smaller than 4 cm, do not require further imaging [1] as they are likely benign; however, in our case, the tumour was causing symptoms, measured 20.8 cm, and malignancy could not be ruled out due to its indeterminate nature noted on imaging.

The summary of cases in Table 2 shows that the median age of patients was 61.7 years. It appeared to be female dominant with a ratio of 1.5 to 1. There was no clear indication of laterality. Over half of the cases presented with symptoms, and over 16% of patients had abdominal symptoms, such as in our patient.

Overall, 70% of cases had normal metabolic workup, and the same was seen in our case. The open surgical approach appeared to be the choice of management in 71% of cases. Lesions were resected by laparoscopic approach in 18 (24.6%) cases, and laparoscopy seemed to be the preferred option for smaller lesions measuring between 3 cm and 12 cm. Having said that, the largest lesion resected laparoscopically was 27 × 17 × 5.5 cm weighing 2 kg [11].

To our knowledge, there is only one other case reported in the literature with anaemia as one of the symptoms of adrenal cavernous haemangioma [12]. Hence, our patient’s presentation with abdominal discomfort and anaemia is atypical. The large adrenal cavernous haemangioma was twice the reported average size (mean size 10.8 cm, mean weight 751.9 g). Similar management was only seen in one other case described in the literature where open left adrenalectomy, distal pancreatectomy, splenectomy, and left radical nephrectomy were performed [13].

Reported radiological signs associated with adrenal haemangiomas are heterogeneous internal structures, speckled calcifications [12], and densely enhanced peripheral rim with patchy enhancement [10]. However, none of these is pathognomonic of adrenal haemangiomas [10]. In our patient, foci of fat, necrosis, and calcification were seen in the left adrenal mass on CT, initially suggestive of myelolipoma. Despite advancements in imaging technology, it is still challenging to differentiate the subsets of adrenal incidentalomas and their likelihood of malignancy via imaging [14,15].

Histologically, adrenal haemangiomas are often described as cavernous, well-encapsulated and localised to the adrenal cortex. They have multiple peripheral vasculature dilatations associated with complex necrotic, calcified, fibrotic, thrombotic and haemorrhagic vascular changes [16]. There are two main histological subtypes of adrenal haemangiomas, namely, cavernous and capillary. The more common cavernous subtype typically comprises infiltrated blood-filled endothelium sinusoids with displaced normal tissues. The capillary subtype is rare and is often composed of tiny areas of submucosal capillaries in loops or lobules [17]. In our case, the lesion showed thrombi-filled dilated blood vessels lined by papillary endothelial hyperplasia with collections of haemosiderophages and foci of dystrophic calcification, which were diagnostic of cavernous haemangioma.

Most reported cases of adrenal cavernous haemangioma were treated surgically, and, indeed, the diagnosis was made frequently following histology. As expected, there are currently no definitive treatment and management guidelines on adrenal cavernous haemangioma. However, The European Society of Endocrinology’s guideline on the management of unilateral adrenal incidentaloma recommends adrenalectomy if there is radiological suspicion of malignancy with or without local structural invasion and clinically relevant hormonal excess [1]. Resection of incidentalomas is not only for diagnostic purposes but can also be therapeutic in achieving symptom relief from compression by the lesion on adjacent viscera. The risk of spontaneous haemorrhage of large lesions is another common indication for early surgical intervention. Otherwise radiological findings of adrenal incidentalomas measuring less than 4 cm in size, homogeneity, and lipid-rich core (<10 HU) are consistent with benign adrenal lesions and managed conservatively [1].

In our case, the patient underwent a major surgical procedure for a benign tumour. This was because of the initial indeterminate mass on radiological imaging and a high risk of biopsy. It was justifiable that the large tumour was resected as it was causing discomfort to the patient and anaemia.

## Conclusions

In summary, adrenal cavernous haemangioma is an extremely rare entity. It typically presents as incidentalomas on imaging. The diagnosis of adrenal cavernous haemangioma is done exclusively by histology. Therefore, diagnostic resection of an adrenal mass as clinically indicated is recommended for lesions larger than 4 cm. Resection may additionally relieve compressive symptoms and avoid potential spontaneous haemorrhage.
